# The cell cycle-regulated DNA adenine methyltransferase CcrM opens a bubble at its DNA recognition site

**DOI:** 10.1038/s41467-019-12498-7

**Published:** 2019-10-10

**Authors:** John R. Horton, Clayton B. Woodcock, Sifa B. Opot, Norbert O. Reich, Xing Zhang, Xiaodong Cheng

**Affiliations:** 1 Department of Epigenetics and Molecular Carcinogenesis, University of Texas MD Anderson Cancer Center, Houston, TX 77030 USA; 2 Department of Chemistry and Biochemistry, University of California, Santa Barbara, CA 93106 USA

**Keywords:** Enzyme mechanisms, X-ray crystallography

## Abstract

The *Caulobacter crescentus* cell cycle-regulated DNA methyltransferase (CcrM) methylates the adenine of hemimethylated GANTC after replication. Here we present the structure of CcrM in complex with double-stranded DNA containing the recognition sequence. CcrM contains an N-terminal methyltransferase domain and a C-terminal nonspecific DNA-binding domain. CcrM is a dimer, with each monomer contacting primarily one DNA strand: the methyltransferase domain of one molecule binds the target strand, recognizes the target sequence, and catalyzes methyl transfer, while the C-terminal domain of the second molecule binds the non-target strand. The DNA contacts at the 5-base pair recognition site results in dramatic DNA distortions including bending, unwinding and base flipping. The two DNA strands are pulled apart, creating a bubble comprising four recognized base pairs. The five bases of the target strand are recognized meticulously by stacking contacts, van der Waals interactions and specific Watson–Crick polar hydrogen bonds to ensure high enzymatic specificity.

## Abstract (web summary)

CcrM is a cell cycle-regulated DNA methyltransferase that methylates an adenine within a specific sequence following replication in the gram negative bacterium *Caulobacter crescentus*. Here the authors present a crystal structure of DNA-bound CcrM that reveals the molecular mechanism leading to sequence-specific methylation.

## Introduction

DNA methylation in bacteria and archaea is common, occurring at the ring carbon C5 of cytosine, the exocyclic amino groups of cytosine (at N4) and adenine (at N6). Genomic DNA adenine methylation within 5’-GANTC-3’ sequences (N = any nucleotide) is an epigenetic mark that controls gene expression in alpha-proteobacteria^1^. Like the mammalian DNA cytosine methyltransferase (MTase) Dnmt1^2^, which is essential for maintenance methylation of hemimethylated CpG dinucleotides at DNA replication forks^3,4^, the DNA adenine MTase (Dam) in *Escherichia coli* and cell cycle-regulated DNA MTase (CcrM) in *Caulobacter crescentus* are responsible for maintenance methylation of GATC or GANTC immediately after replication, respectively^5,6^. Whereas Dam molecules from *E. coli* and the bacteriophage T4 were structurally characterized in complex with cognate and non-cognate DNA^7–10^, no structures for CcrM are known.

Many sequence-specific DNA binding proteins exercise their effects by binding to specific genomic regions. Nucleotide modifying enzymes, such as DNA MTases that methylate the target nucleotide (cytosine or adenine) within a specific DNA sequence, face the obstacle of having to bring the intrahelical target nucleotide into a concave catalytic pocket. Using the base flipping process, DNA MTases rotate the target nucleotide along the flanking phosphodiester bonds such that the flipped nucleotide projects into the active-site pocket where the catalytic residues reside^11^. However, prior studies of CcrM and orthologs suggested that CcrM utilizes a previously unknown DNA recognition mechanism to yield discrimination for the recognition sequence^6,12–15^. Here, by means of X-ray crystallography, we show that a CcrM dimer binds DNA by opening up double stranded DNA at the recognition site, a DNA recognition mechanism that likely contributes to the enzyme’s sequence discrimination.

## Results

### CcrM forms a dimer

**Fig. 1:**
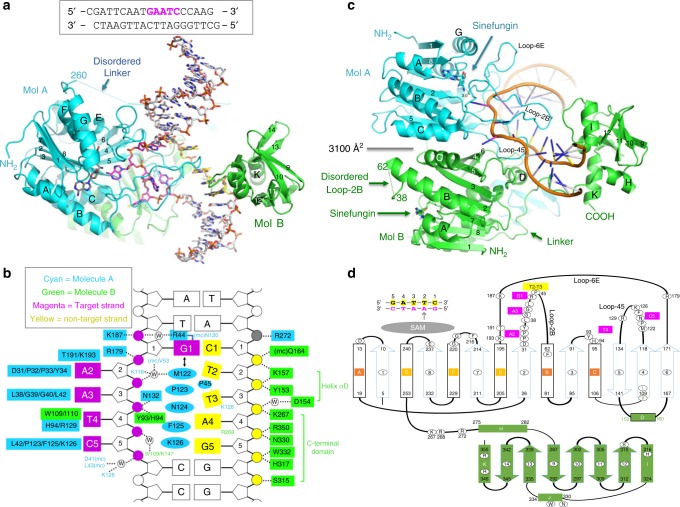
Structure of CcrM-DNA complex. **a** The MTase domain of molecule A (in cyan) and the C-terminal domain of molecule B (in green) collaborate for the DNA binding. **b** Schematic of CcrM-DNA interactions: mc, main-chain-atom-mediated contacts: w, water-mediated hydrogen bonds. DNA bases in magenta represent the target strand, while non-target strand DNA bases are in yellow. Molecule A residues are in cyan, molecule B residues in green. **c** CcrM forms a dimer (Mol A in cyan and Mol B in green). The labels of secondary structural elements are according to panel **d**. **d** Schematic diagram of CcrM secondary structures, with N-terminal MTase domain (cyan lines for strands and black lines for helices) and C-terminal DNA binding domain (green). Helices are labeled from A to H and strands are labeled as 1 to 14

We used an 18-base-pair oligonucleotide (plus a 5’-overhanging cytosine on one strand and a guanine on the other strand) containing a non-centrally located GAATC site for co-crystallization with CcrM (Fig. 1a). We crystallized the CcrM-DNA complex in the presence of sinefungin (a methyl donor analog) in space group *P*2_1_2_1_2_1_ and determined the structure to the resolution of 2.34 Å (Supplementary Table 1). The DNA-protein contacts are concentrated on the five base pairs of the recognition site by a CcrM dimer (Fig. 1a, b).

The DNA-bound form of CcrM contains two molecules (A and B) (Fig. 1a, c). Each molecule comprises an N-terminal MTase domain (residues 1–260) of an 8-stranded β-sheet (β1-β8) with three helices (αA, αB, αC, and αE, αF, αG) located on each side of the sheet, resulting in an open αβα sandwich^16,17^. The C-terminal domain (residues 271–358) is connected to the N-terminal MTase domain via a 10-residue linker (amino acids 261–270) (Fig. 1d). The dimer interface with an area of ~3100 Å^2^ is mainly mediated by the MTase domains with two active sites (indicated by the binding of sinefungin) facing opposite directions (Fig. 1b). The two monomers contribute unevenly to DNA binding. The MTase domain of molecule A (in cyan) makes most of the contacts to the target strand (in magenta) and catalyzes methyl transfer, while the C-terminal domain of molecule B (in green) is solely responsible for the binding of the non-target strand (in yellow; Fig. 1a, b). Each molecule has a disordered loop when the corresponding function is not used. In molecule A, the C-terminal domain was not involved in direct DNA contact and the linker between the two domains is disordered (Fig. 1a). In molecule B, the active site is faced away from the DNA and part of the corresponding active-site loop, Loop-2B (between strand β2 and helix αB), is disordered (Fig. 1b).

### Distortion of DNA conformation

**Fig. 2:**
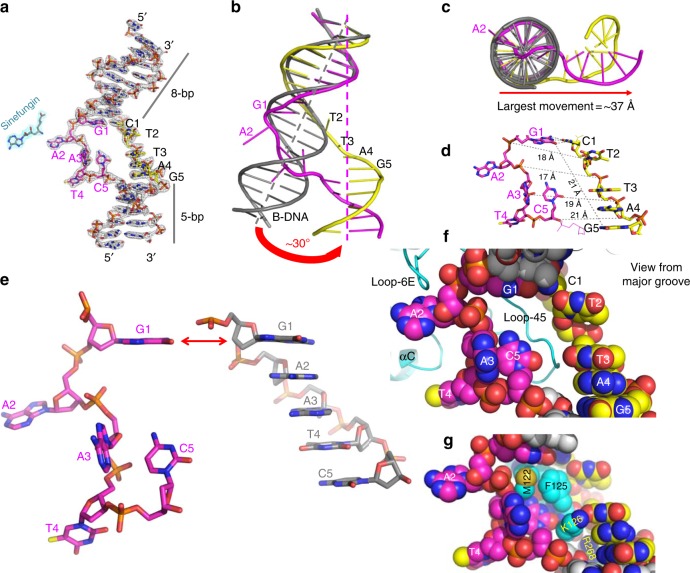
CcrM-bound DNA conformation. **a** Electron density 2Fo-Fc, contoured at 2*σ* above the mean, is shown for the entire DNA and sinefungin (see Supplementary Fig. 2b). **b**, **c** Superimposition of B-DNA (gray) and CcrM-bound DNA molecule (colored). **d** Increased inter-strand phosphate-to-phosphate distance and increased base step distance between two guanines G1 and G5. **e** Structural comparison of the target sequence in CcrM-bound form (left) and B-DNA form (right) by superimposing G1 nucleotide. **f**, **g** View from DNA major groove, with residues of Loop-45 (**f**) from the minor groove in a space filling model (**g**)

The CcrM-bound DNA molecule undergoes dramatic distortions at the 5-bp recognition site while the outer sequences at both ends maintain B-form (Fig. 2a). We generated a standard B-DNA of the same sequence and superimposed it onto the CcrM-bound DNA (Fig. 2b). First, the bound DNA molecule is kinked between two thymine residues (T2 and T3) of the non-target strand and bent ~30° (Fig. 2b), resulting in the largest movement of ~37 Å towards one end of the DNA molecule (Fig. 2c). Second, the two strands are pulled apart by increasing the inter-strand phosphate-to-phosphate distance up to 20.9 Å from that of 17.5 Å in B-DNA, and unwound by increasing the base step distance to 21.2 Å (between two guanines G1 and G5), from that of 13.7 Å in B-DNA (Fig. 2d). Subsequently, four out of the five base pairs within the recognition sequence are disrupted (A2:T2 to C5:G5), with the four bases of the target strand (A2 to C5) being repositioned into new locations and being completely out-of-stacking with neighboring bases (Fig. 2e). The nucleotides A2 and T4 are flipped completely out of the helix by a near 180° torsional rotation about the backbone. Similarly, the nucleotide A3 is rotated ~90° along its flanking phosphodiester bonds and appears to be trapped in an incomplete flipping position. In contrast, the C5 nucleotide is located in an intra-helical position, but perpendicular to its initial position in the B-DNA. While the extrahelical positioning of the target A2 base is expected based on numerous studies with other DNA MTases^11,18,19^, the dramatic distortion along most of the recognition sequence is thus far unique to CcrM. These distortions create a bubble in the DNA with the protein Loop-45 (between strands β4 and β5) of molecule A approaching from the minor groove and occupying the open space between the two strands (Fig. 2f, g). Currently, we do not know how these events unfold temporally leading to the observed distortions.

### DNA strand separation

**Fig. 3:**
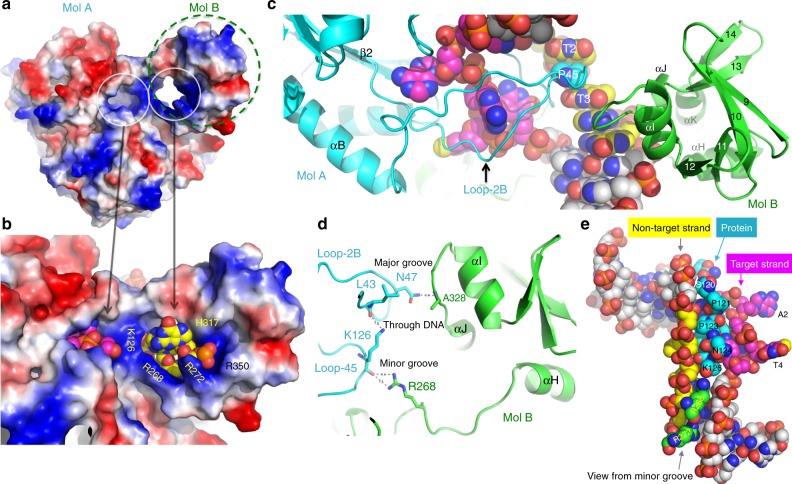
DNA strand separation by CcrM. **a**, **b** CcrM dimer contains two closed holes for the two separated DNA strands. The surface charge at neutral pH is displayed as blue for positive, red for negative, and white for neutral. **c** Pro45 of Loop-2B wedges into thymine bases T2 and T3 of the non-target strand. **d** A network of protein-protein interactions centered on DNA major groove, DNA minor groove, and through the space between the two strands including three pairs of polar interactions involving side chains of Asn47, Lys126, and Arg268 and the main-chain carbonyl oxygen atoms of Ala328, Leu43, and Lys126, respectively. **e** View from DNA minor groove side, with residues of molecule B (cyan) and molecule A (green) occupying the space between two strands

The CcrM dimer, particularly the N-terminal domain of molecule A and C-terminal domain of molecule B, interacts to form two doughnut shaped holes lined with basic residues (Fig. 3a), sufficient for holding the two separate strands apart (Fig. 3b). There are inter-molecular and intra-molecular interactions (between A and B) across the DNA both in the major and minor grooves, as well as through the open space between the two separated strands that likely confer stability to this unusual protein-DNA complex. In molecule A, the 30-residue Loop-2B approaches DNA from the major groove with the tip of the loop, Pro45, wedging in-between the two thymine bases T2 and T3, causing a kink in the non-target strand and displacing thymine T2 to the border of the double helix (Fig. 3c). The Loop-2B is held in place through interactions with the C-terminal domain of molecule B (Fig. 3d). From the DNA minor groove side, Loop-45 provides seven residues (Ser120-Lys126) occupying the space between the two DNA strands (Fig. 3e), with the main-chain carbonyl oxygen of Lys126 interacting with Arg268 of molecule B on the minor groove side (Fig. 3d). In addition, Loop-2B and Loop-45, which approaches the DNA from opposite directions and penetrate into the DNA, forms interactions passing through the DNA strands (Fig. 3d). Thus, the protein-DNA interface is comprised of a network of stabilizing interactions involving three segments of the CcrM dimer, Loop-45 and Loop-2B of molecule A and C-terminal domain of molecule B (Fig. 3d).

### Base specific recognition

**Fig. 4:**
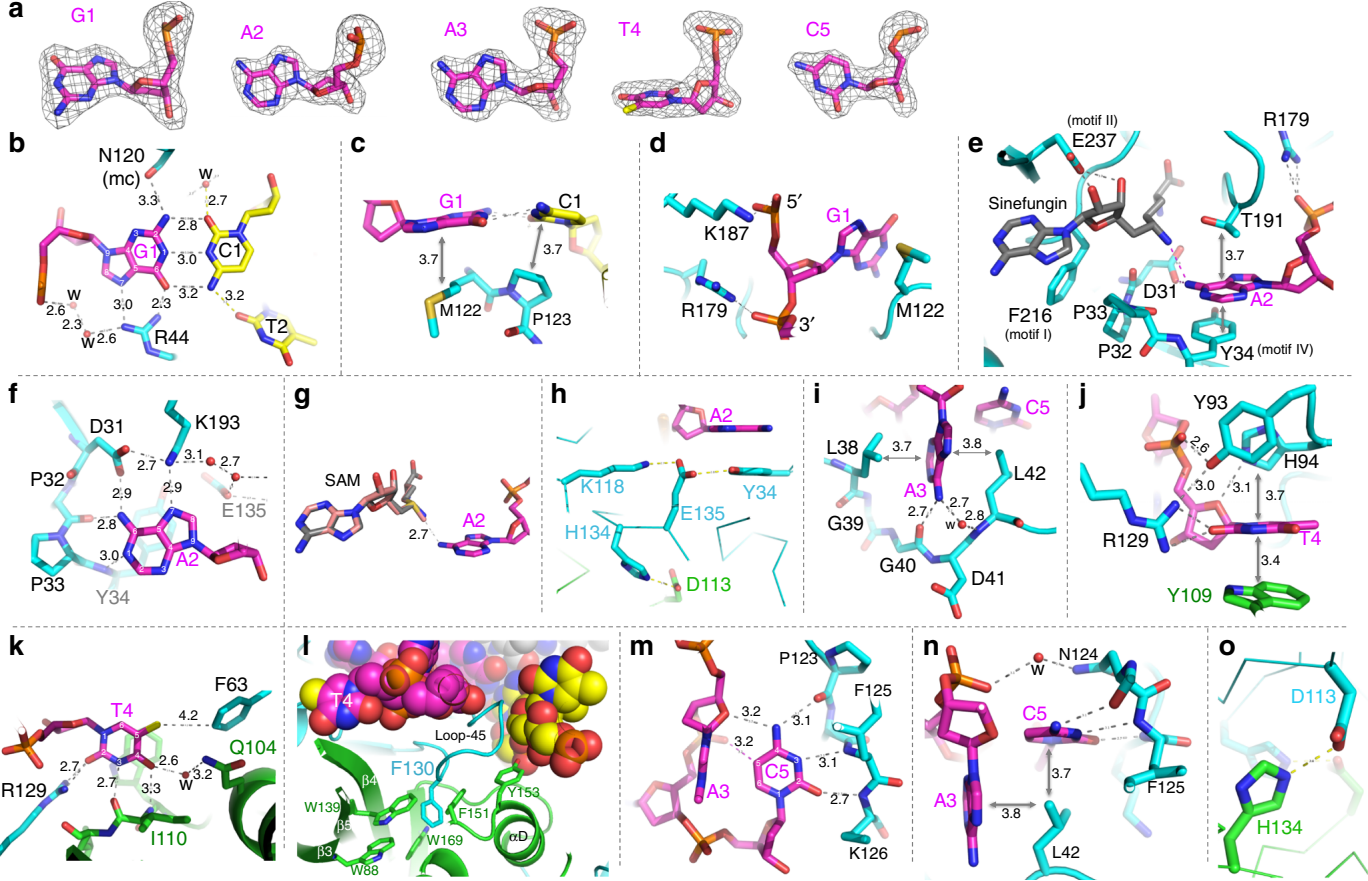
Base specific recognition of target strand. **a** The omit electron density (Fo-Fc), contoured at 5.0*σ* above the mean, for omitting each individual nucleotide of the target sequence. **b**, **c** Interactions with G1:C1 base pair. Interatomic distances are shown in angstroms. **d** Arg179 and Lys187 of Loop-6E interact with phosphate groups flanking G1. **e**, **f** Interactions with the target adenine A2 in the active-site pocket. **g** Superimposition of methyl donor SAM onto sinefungin. **h** A network of interactions involving Lys118, Glu135, and Tyr34 in stabilization of target base A2. **i** Interaction with the variable position of A3 in GANTC (N = any nucleotide). **j**, **k** Interactions with thymine T4. **l** CcrM dimer interaction involving Phe130 of molecule B (cyan) and an aromatic cage of molecule A (green). **m**, **n** Interactions with cytosine C5. **o** Two pairs of H134-Asp113 interactions in the dimer interface

Four loops, three long and one short, in molecule A after the carboxyl ends of strands β2 (Loop-2B), β3 (Loop-3C), β4 (Loop-45), and β6 (Loop-6E) (as shown in Fig. 1d) provide most of the functionally important residues in recognizing the five bases of the target strand (Fig. 4a) [Molecule B is involved in recognition of thymine T4, see below]. Loop-2B (residues 31–61) recognizes the first three bases of the target sequence, guanine G1, adenine A2, and adenine A3. Loop-45 (residues 119–133) provides interactions for binding the two pyrimidines, T4 and C5, while the short Loop-3C (residues 92–94) and Loop-6E (residues 172–194) supply additional interactions for thymine T4 (Tyr93 and His94), target adenine A2 (Thr191 and Lys193), and DNA backbone phosphate groups flanking guanine G1 (Arg179 and Lys187; Fig. 1b).

As noted above, Pro45 of Loop-2B intercalates into the non-target strand (Fig. 3c), whereas the residues N-terminal to Pro45 recognize the first three bases of the target sequence. The first G1:C1 base pair stays intra-helical and maintains Watson–Crick hydrogen bonds (Fig. 4b). In accordance with the most common mechanism for guanine recognition^20–22^, Arg44 forms bidentate hydrogen bonds with the *O6* and *N7* atoms of the G1 base. In addition, Met122 and Pro123 of Loop-45 provide van der Waals contacts to the G1:C1 base pair, from which the second A2:T2 base pair would be located in a normal B-DNA molecule (Fig. 4c). Substitution of GACTC by AACTC in dsDNA results in a 10^6^–10^7^-fold loss in specificity for CcrM^14^, which is most likely driven by the loss of G1-Arg44 interaction. The corresponding Gua-Arg interaction for the last C:G base pair in GATC by *E. coli* Dam is also a sequence discriminatory contact^8^.

Like other structurally characterized DNA MTases^11,18,19^, the target adenine A2 is flipped out and inserted into the active-site pocket where sinefungin is bound (Fig. 4e). The adenine is surrounded by the DPPY motif, a catalytically active sequence motif conserved among amino MTases^23^, and is stacked in-between Tyr34 and Thr191 (Fig. 4e). The polar groups of the target adenine ring (*N1*, *N6*, and *N7*), that normally form the Watson–Crick pair with thymine and/or interaction with protein in the major groove, are now involved in hydrogen bonds with the main-chain amide nitrogen of Tyr34 (interacting with *N1* atom), the side chain carboxylate oxygen of Asp31 and main-chain carbonyl oxygen of Pro32 (interacting with *N6* amino group), and side-chain of Lys193 (interacting with *N7* atom) (Fig. 4f). This pattern of hydrogen bonding defines the specificity for adenine in the active-site binding pocket, and positions the target *N6* atom in line with the methyl group and sulfur atom of *S*-adenosyl-l-methionine (SAM) (Fig. 4g). This linear arrangement, comprising the nucleophile, the methyl group and the leaving thioester group in the transition state, is required for the S_N_2 reaction mechanism used by SAM-dependent MTases^17^.

The next base is variable within the recognition sequence, and adenine A3 used in the crystallization is stacked in-between two hydrophobic residues, Leu38 and Leu42 (Fig. 4i). However, unlike the target adenine A2, the hydrogen bonds are limited to the exocyclic amino group *N6* of A3 by the main-chain carbonyl oxygen of Gly40 and a water molecule (Fig. 4i). We note that two rigid proline residues (Pro32 and Pro33), which have the least conformational freedom, are used to configure the specific A2 binding pocket, whereas two flexible glycine residues (Gly39 and Gly40), which can adopt many different main-chain conformations, are used to define the variable position of the recognition sequence.

Thymine T4 is recognized by the combinatory effect of molecule A (Tyr93, His94 and Arg129 in cyan) and molecule B (Tyr109 and Ile110 in green) (Fig. 4j, k). The base T4 is sandwiched between Tyr109 (molecule B) and Tyr93 and His94 (molecule A). In addition, the two residues of molecule A interact with the 5’ phosphate group and the ribose, respectively (Fig. 4j). The polar groups of the T4 ring (*O2*, *N3*, and *O4*) are involved in hydrogen bonds with Arg129 (molecule A) and the main-chain carbonyl oxygen and amide nitrogen atoms of Ile110 (molecule B), respectively (Fig. 4k). In addition, the methyl group at the ring *C5* position, which is unique to thymine, makes a van der Waals contact with Phe63 of molecule A (Fig. 4k). As noted above, CcrM has a particularly large dimer interface; and Phe130, the residue immediately after Arg129, demonstrates an example of dimer interaction involving an aromatic cage where Phe130 is inserted (Fig. 4l).

The last base, cytosine C5, is trapped between the residues of Loop-45 and the target DNA strand (Fig. 4m, n). The polar groups of the C5 ring (*O2*, *N3*, and *N4*) that normally form the Watson-Crick pair with guanine are now involved in hydrogen bonds with three main-chain atoms, i.e., the main-chain amide nitrogen atoms of Lys126 and Phe125, and the main-chain carbonyl oxygen atom of Pro123 (Fig. 4m). In addition, the C5 base makes two intra-molecular interactions within the same strand with the ribose oxygen of adenine A3 (via the *N4* amino group) and the phosphate oxygen atom of thymine T4, via the ring *C5* atom forming a H–C•••O type hydrogen bond^24^ (Fig. 4m). Presence of a methyl group at the *C5* position (either by cytosine methylation or cytosine-to-thymine substitution) would sterically obstruct the cytosine specific conformation. In sum, like Watson-Crick base pairs in the dsDNA, the base pairing pattern, van der Waals and pi–pi interactions between CcrM and bases of the recognition sequence provide the driving force for the different base conformations observed. In an alanine mutation study of 20 chosen residues of CcrM, four mutants resulted in more than 100-fold reduction of methylation activity^25^; they are K118A (involved in a relay interaction in stabilizing A2; Fig. 4h), R129A (recognition of T4; Fig. 4j), H134A (dimer interaction; Fig. 4o), and R179A (DNA phosphate interaction between G1 and A2; Fig. 4d).

### Interaction with the non-target strand

**Fig. 5:**
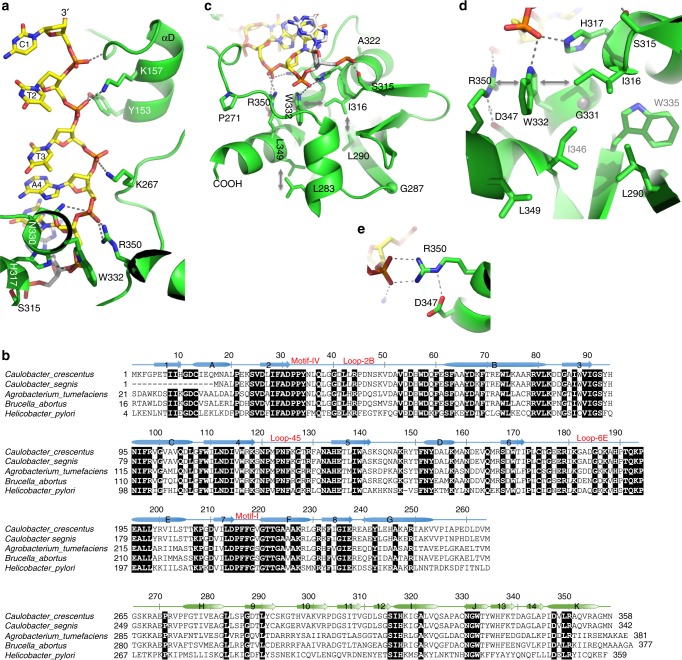
Sequence conservation of CcrM orthologs. **a** Molecule B (helix αD and C-terminal domain) provides almost all DNA phosphate interactions along the non-target strand. **b** Sequence alignment of five CcrM orthologs: *Caulobacter crescentus* (NC_002696), *Caulobacter segnis* (NC_014100.1), *Agrobacterium tumefaciens* (NC_003062), *Brucella abortus* (AF011895), and *Helicobacter pylori* (AGR62562). Invariant residues are marked with white letters on black background. **c-e** Structural features of invariant residues in the C-terminal domain. **c** Pro271 and Gly287 are structural residues located in the linker loop and the beginning of strand β9. **d** Leu283, Leu290, Ile316, Gly331, Trp332, and Leu349 are part of the hydrophobic core that supports the integrity of the C-terminal domain. **e** Asp347 and Arg350 form an electrostatic interaction

The C-terminal domain is folded as six antiparallel strands (β9-β14) with three short helices (αH, αI, and αK) and one 3_10_ helix (αJ) packed against one side of the twisted β-sheet (Fig. 1). The Vector Alignment Search Tool^26^ revealed that the C-terminal domain of CcrM resembles that of the eukaryotic SAND domain (named after Sp100, AIRE-1, NeuP41/75, and DEAF-1) that shares structural similarity to the PWWP domain of mammalian DNA MTase Dnmt3b^27,28^ (Supplementary Fig. 1). Both SAND and PWWP domains were demonstrated to bind DNA nonspecifically^27,28^. Moreover, the C-terminal domain of CcrM was suggested to be involved in DNA binding^29^ and deletion of the domain results in loss of enzymatic activity^15^. Indeed, in the current DNA-bound CcrM structure, molecule B provides almost all DNA phosphate contacts of the non-target strand, with interactions mediated by the C-terminal domain concentrated on the four 5’ phosphates surrounding the recognition sequence (Figs. 1b and 5a). Furthermore, the C-terminal domain of molecule B is involved in the crystal packing contacts with two neighboring molecules (Supplementary Fig. 2a), which might contribute to the current positioning of the domain.

Sequence alignment of representative CcrM orthologs indicates that the N-terminal MTase domain is relatively well conserved (~46% identity), whereas the C-terminal domains share much less identity (~14%) (Fig. 5b). Nevertheless, invariant residues are scattered throughout the entire C-terminal domain, including those involved in structural integrity or DNA phosphate binding (Ser315, His317, Asn330, Trp332, and Arg350) (Fig. 5c-e). A mutation of Trp332-to-alanine (W332A) produced an inactive mutant^15^, whereas in a separate study the four alanine mutations S315A, H317A, N330A, and R350A showed ~90% reduced methylation activity on dsDNA but no effect on SAM binding^29^, though S315A had lost DNA binding activity^30^. In *C. crescentus*, S315A and W332A caused severe defects in cell viability, cell division and morphology, exhibiting filamentous bacterial growth^30^. Besides direct DNA phosphate contact, Trp332 is sandwiched between Arg350 and Ile316 via van der Waals contacts (Fig. 5d), providing an additional support for the local stability. The strand-separated structure we observed is probably the final product of the substrate-recognition pathway conducted by CcrM. We do not know whether the C-terminal domain participates in the initial processive scanning of DNA to locate a cognate site^29^. In the related *Escherichia coli* Dam MTase, the same set of protein residues can switch, from an electrostatic interaction with the DNA backbone in a nonspecific complex, to a specific binding mode with DNA base pairs in the cognate complex^8^.

### Comparison with other dimeric MTases

**Fig. 6:**
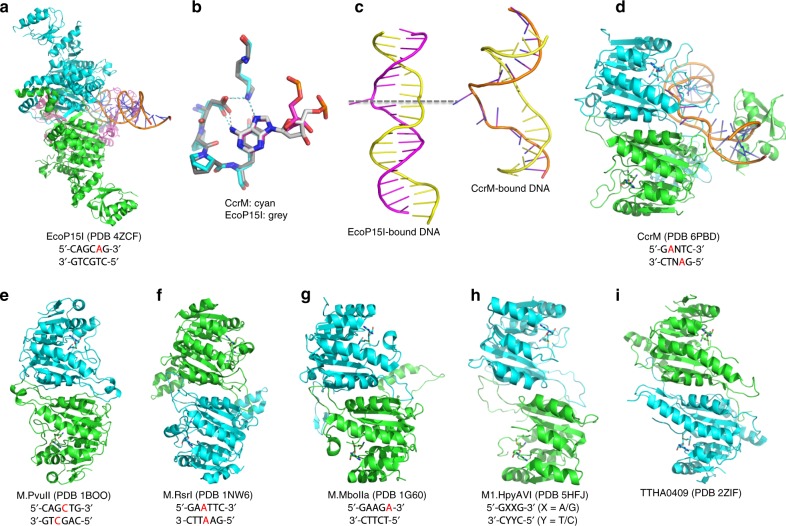
Conserved dimer formation in β-class amino MTases. **a** EcoP15I (PDB 4ZCF). **b** Superimposition of EcoP15I and CcrM results in a conserved active-site configuration for adenine. **c** Two very different DNA conformations in the EcoP15I-bound (left) and CcrM-bound (right). Except for the target adenine, the EcoP15I-bound DNA conformation has intact intra-helical bases. **d** CcrM (PDB 6PBD). **e** M.PvuII (PDB 1BOO) on cytosine–N4 methylation. **f** M.RsrI (PDB 1NW6). **g** M.MboIIA (PDB 1G60). **h** M1.HpyAVI from *Helicobacter pylori* (PDB 5HFJ). **i** TTHA0409 from *Thermus thermophilus* HB8 (PDB 2ZIF)

Based on the sequential order of conserved sequence motifs, particularly sequences for binding of the methyl donor SAM (motif I: FxGxG) and for catalysis (motif IV: (D/N)PP(Y/F/W), CcrM is a β-class MTase^23^ (Fig. 5b). Like CcrM, other structurally characterized β-class amino MTases (acting on DNA adenine-N6 and cytosine-N4), in the presence or absence of bound substrate, can form dimers (Fig. 6). Comparing the different structures of the β-class MTases, similarities suggest an evolutionary link between homodimers recognizing a palindromic sequence (such as M.PvuII^31^ and M.RsrI^32^), and those recognizing an asymmetric sequence (such as CcrM, EcoP15I^19^, M.MboIIa^33^ and a DNA N6-adenine methyltransferase from Helicobacter pylori^34^) (Fig. 6). Superimposition of EcoP15I and CcrM results in a conserved active-site configuration for the target adenine (Fig. 6b). Except for the target adenine, the EcoP15I-bound DNA conformation has intact intra-helical bases and the enzyme-bound DNA molecules exhibited two very different conformations (Fig. 6c). We note that CcrM is active on both ds and ssDNA containing G**A**nTC, but not on ssRNA^14^ (Supplementary Fig. 3), whereas a recently characterized β-class non-specific adenine MTase (M.EcoGII) is active on ds and ss DNA, ss RNA, and ds DNA/RNA hybrid^35^. Thus far, screenings of CcrM with single-stranded oligonucleotides have failed to yield crystals. Additional data will be required to uncover the mechanism of single stranded DNA recognition by CcrM.

## Discussion

Here we describe the interaction of *C. crescentus* CcrM with its dsDNA substrate, resulting in a separation of two DNA strands at the recognition site, in agreement with CcrM activity on both ds and singled-stranded DNA in vitro^14^. As expected, CcrM uses the base flipping mechanism to project the target nucleotide out of the double helix and into the active site pocket. Base flipping is a common mechanism that is widely used by nucleotide-modifying enzymes^36^, sometimes in conjunction with other DNA distortions including kinking^37^ and helix unwinding^38^. What has not been seen before is the consolidation of multiple significant DNA distortions into a single protein-DNA binding event by a relatively small enzyme. Upon sequence recognition, CcrM kinks and unwinds the DNA molecule, intercalates into the minor groove and promotes eversion of four nucleotides of the target strand. The binding and recognition of displaced nucleotides may be critical for the level of discrimination for the recognition sequence by CcrM^14,15^.

## Methods

### Purification of CcrM

The *C. crescentus* CcrM gene was cloned out of the pET-IMPACT plasmid^39^ into a modified pET-28 based expression vector using EcoRI and NdeI restriction sites, to generate a N-terminal His-tag fusion protein (pXC2121). Subsequently, it was transformed into the BL21(DE3) C + *Escherichia coli* strain. Bacterial cultures using LB broth were inoculated from an overnight starter culture at 37 °C and grown until a culture density of *A*_600 nm_ ~1, at which the temperature is shifted to 16 °C and CcrM expression induced with addition of 0.4 mM isopropyl β-*d*-1-thiogalactopyranoside (IPTG); cultures were allowed to proceed for an additional 16 h. The BL21 cells were lysed by sonication in buffered solution [20 mM HEPES (pH 8.0), 300 mM NaCl, 10% glycerol, 0.5 mM tris (2-carboxyethyl) phosphine (TCEP)] and 1 mM phenylmethylsulfonyl fluoride (PMSF)]. The lysed sample was clarified by centrifugation at 25,000 rpm for 2 h at 4 °C and passed through a 3.1 μm filter (Thermo Scientific Titan3 Filter). The supernatant was collected and subjected to a three-column chromatography conducted on a GE Healthcare ÄKTA purifier or a BIO-RAD NGC™ system. The sample was brought to a final concentration of 60 mM imidazole before loading onto a 5-ml HisTrap Ni-column (GE Healthcare) at flow rate of 1 ml/min. The column was washed with 60 ml of the buffered solution containing 60 mM imidazole at flow rate of 2.5 ml/min. The His-tagged protein was eluted with an imidazole gradient from 60 mM to 300 mM with the peak maximum at 160 mM. Eluted fractions were pooled and diluted into a buffer with lower pH with a final concentration of 50 mM NaCl, 20 mM HEPES, pH 6.8, 5% glycerol, and 0.5 mM TCEP. The protein was further purified by 5-ml HiTrap Q-SP columns (GE Healthcare) connected in tandem^40^. After the sample was loaded the Q column was physically removed and the SP column was washed with 50 ml of the low pH buffer followed by a 100 ml linear NaCl gradient from 50 mM to 1 M NaCl at 1 ml/min. CcrM was eluted in two distinct peaks at ~360 mM and 700 mM NaCl. The two peaks were pooled separately and the high salt peak was used for crystallization successfully.

The pooled fractions were concentrated to ~2 ml using Amicon Ultra centrifugal filters (10 kDa MWCO) and loaded onto a Superdex 200 16/60 (GE Healthcare) column equilibrated in the higher pH buffered solution. CcrM eluted from the sizing column as a dimer. The protein was collected as a single peak and concentrated to 50 mg/ml (~140 μM in monomer concentration) in 20 mM HEPES pH 8.0, 300 mM NaCl, 5% glycerol, 0.5 mM TCEP.

### Crystallography

We mixed the purified protein with an approximately equal molar ratio of monomer to double-stranded DNA duplex (~56 μM) (Supplementary Table 2) and a 5× molar ratio sinefungin (Sigma) for co-crystallization. An Art Robbins Gryphon Crystallization Robot was used to set up sitting drop at 19 °C by mixing 0.2 µl of the complex with an equal volume of well solution. Large individual crystals grew in 0.1 M HEPES pH 7.8, with 10% (w/v) polyethylene glycol 8000 and 3% (w/v) polyethylene glycol 6000. These crystals formed overnight and were stable for ~5 days after which they began to dissolve. CcrM screens with varying ratios of DNA (0.5, 0.75, 1, and 2) resulted in no crystal formation (1:0.5), fewer crystals (1:0.75) than that of 1:1, and crystals deteriorated quicker (in less than 5 days) with a 1:2 ratio.

Crystals selected for X-ray data collection were quickly frozen after increasing the polyethylene glycol concentration of the crystallization solution to >30%, captured in a nylon loop, and immersed into liquid nitrogen. Data was collected at the beamline 22-ID of SER-CAT of Advanced Photon Source at 1.0 Å wavelength by rotating a mounted crystal a total of 400° in 0.25° increments per image. HKL2000 was utilized for data reduction and scaling^41^. Crystals grew in the *P*2_1_2_1_2_1_ space group with the best diffracting power having a smaller unit cell particularly along the **a** axis (possibly the result of dehydration in the cryosolution containing a higher concentration of PEG) (Supplementary Table 2) with an asymmetric crystallographic unit containing one CcrM dimer with one DNA duplex.

The molecular replacement method gave initial phasing. The PHYRE2 server^42^ was utilized for generation of a model of the N-terminal CcrM MTase domain based on the structure of M.MboIIa (PDB code 1G60); all loops in this model were removed before using it as a search model in the PHENIX PHASER module^43^. In addition, an 18-mer B-DNA containing the sequence used in the crystallization was generated by the make-na server (http://structure.usc.edu/make-na/server.html) and used as a secondary search model. Two molecules of the protein model could be found in positions so that they formed a dimer similar to that of other β-class amino MTases with one DNA molecule appearing to clash the dimer. While lower resolution datasets (~3 Å) gave electron density maps not so interpretable, a 2.34 Å dataset gave maps that allowed loop building and model correction. Later refinements using PHENIX^44^ revealed the correct DNA structure (as the molecular replacement solution contained part of a neighboring DNA in another asymmetric unit) and allowed model building of linker and the C-terminal domains in each monomer. COOT^45^ was used for model building and corrections between refinement rounds. Structure quality was analyzed during PHENIX refinements and later validated by the PDB validation server. Molecular graphics were generated using PyMol (Schrödinger, LLC). We note that we obtained crystals with hemi-methylated DNA under similar conditions. However, the structure did not show any difference with unmodified DNA.

### Reporting Summary

Further information on research design is available in the Nature Research Reporting Summary linked to this Article.

## Supplementary Information

Supplementary Information

Peer Review File

Reporting Summary

## Data Availability

The data that support the findings of this study are available from the corresponding authors upon request. The X-ray structure (coordinates) and the source data (structure factor file) of CcrM with bound DNA have been submitted to the PDB under accession number 6PBD.
