# Supplementary material for: The cell cycle-regulated DNA adenine methyltransferase CcrM opens a bubble at its DNA recognition site
Source: Nat Commun. 2019 Oct 10;10:4600. doi: 10.1038/s41467-019-12498-7 (PMC6787082; doi:10.1038/s41467-019-12498-7)
Supplement: Supplementary file 1 — Supplementary Information [file 41467_2019_12498_MOESM1_ESM.pdf]

Supplementary Information

Horton et al.

The cell cycle-regulated DNA adenine methyltransferase CcrM opens a bubble at its DNA recognition site

John R. Horton[1,3], Clayton B. Woodcock[1,3], Sifa B. Opot[1], Norbert O. Reich[2], Xing Zhang[1,4*], Xiaodong Cheng[1,4*]

[1]Department of Epigenetics and Molecular Carcinogenesis, University of Texas MD Anderson Cancer Center, Houston, TX 77030, USA

[2]Department of Chemistry and Biochemistry, University of California, Santa Barbara, CA 93106, USA

[3]These authors contributed equally: John R. Horton, Clayton B. Woodcock.

[4]These authors jointly supervised this work: Xing Zhang, Xiaodong Cheng

* e-mail: xzhang21@mdnderson.org; xcheng5@mdanderson.org

3 Supplementary Figures

2 Supplementary Tables

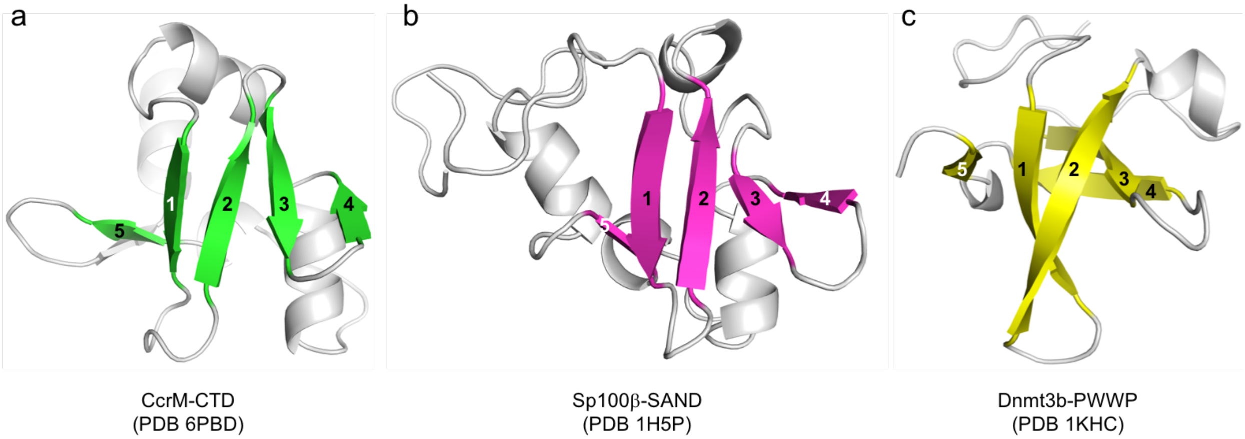

Supplementary Figure 1. Structural comparison of (**a**) the CcrM C-terminal domain, (**b**) the Sp100b SAND domain, and (**c**) the Dnmt3b PWWP domain. The structural equivalent of five anti-parallel β-strands are colored. The result of a VAST search indicated similarity between CcrM C-terminal domain and the Sp100b SAND domain (aligned residues=46, score=7.3, p-value=0.012, and root-mean-squared-deviation=1.7 Å).

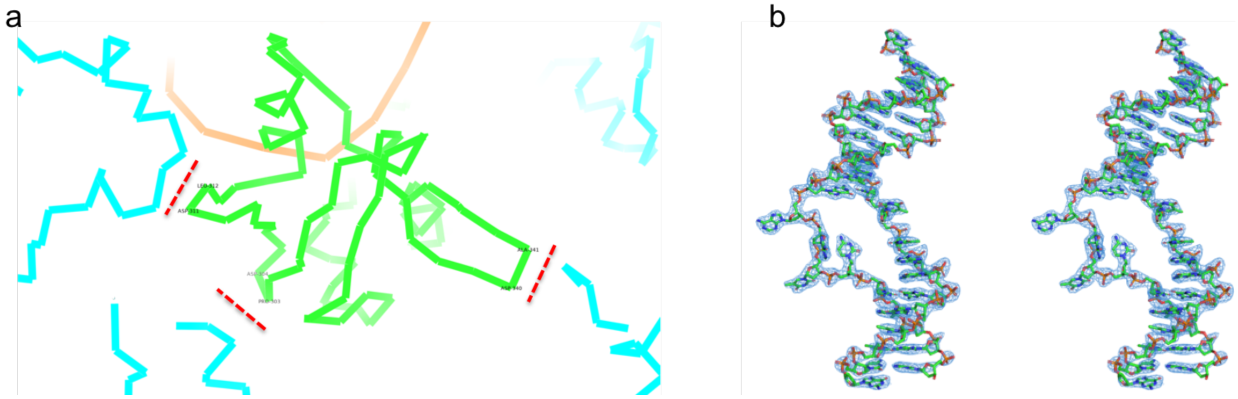

Supplementary Figure 2. (**a**) Three loop regions of the C-terminal domain of Molecule B (green) are in contact with two neighboring molecules A (in cyan) within the crystal lattice. These regions are not involved in interactions with DNA phosphate backbone (orange). Dashed red lines indicates the contact interfaces. (**b**) A stereo image of electron density 2Fo-Fc, contoured at 2σ above the mean, is shown for the entire DNA molecule.

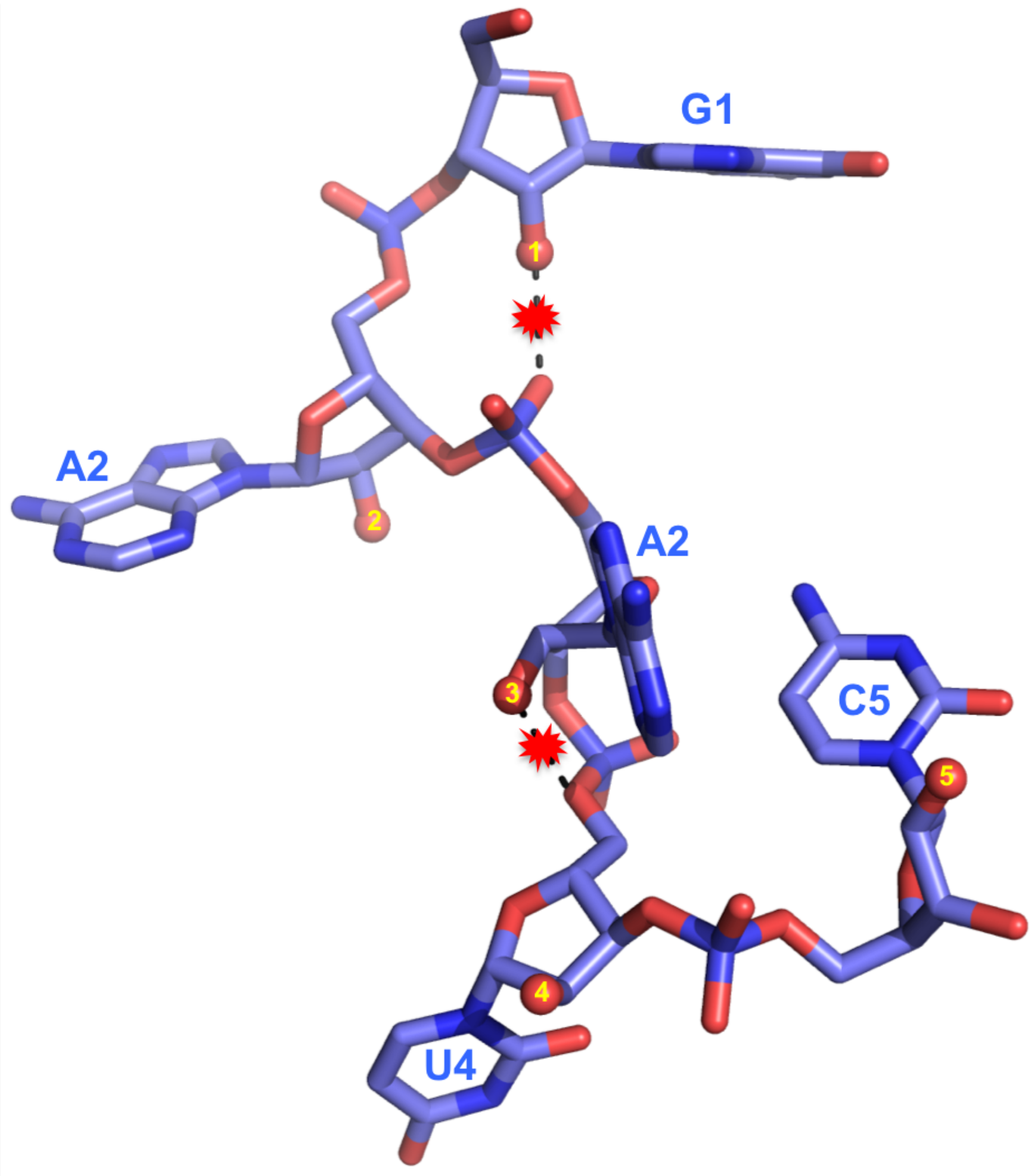

Supplementary Figure 3. Model of ssRNA. Assuming the 5-nt recognition sequence (5'-GAAUC-3') in ssRNA adopts the same conformation as the target strand in the CcrM-bound dsDNA, modeling a 2'-hydroxyl group (OH) onto sugar ribose (as happens in RNA) potentially results in intra-strand repulsion (as indicated by red stars) between the G1 OH group and one of the A3 phosphate oxygen atoms or between the A3 OH group and the U4 O5' oxygen atom. CcrM can accommodate the other three OH groups at A2, A3, and C5.

Supplementary Table 1. Summary of X-ray data collection from SERCAT beamline (22-ID) at a wavelength of 1 Å

CcrM	PDB 6PBD
DNA (5'-3')	CGATTCAATGAATCCCAAG
(3'-5')	CTAAGTTACTTAGGGTTCG
**Data Collection**	
Space group	$P2_12_12_1$
Cell dimensions (Å)	67.68, 117.94, 119.60
α, β, γ (°)	90, 90, 90
Resolution (Å)	41.68-2.34 (2.43-2.34) *
[a] R_{merge}	0.113 (0.766)
R_{pim}	0.035 (0.398)
$CC_{1/2}$, CC	(0.753, 0.927)
[b] $\langle I/\sigma I \rangle$	19.7 (1.9)
Completeness (%)	99.7 (97.7)
Redundancy	10.2 (4.0)
Observed reflections	415,883
Unique reflections	40,730 (3929)
**Refinement**	
Resolution (Å)	2.34
No. reflections	39,041
[c] R_{work} / [d] R_{free}	0.175 / 0.207
No. Atoms	
Protein	5203
DNA	770
Sinefingin	54
Solvent	163
B Factors ($Å^2$)	
Protein	55.6
DNA	69.6
Sinefingin	43.8
Solvent	47.5
**R.m.s. deviations**	
Bond lengths (Å)	0.002
Bond angles (˚)	0.5

* Values in parenthesis correspond to highest resolution shell;

[a] $R_{merge}=\Sigma|I-\langle I\rangle|/\Sigma I$, where I is the observed intensity and $\langle I\rangle$ is the averaged intensity from multiple observations.

[b] $\langle I/\sigma I\rangle$ = averaged ratio of the intensity (I) to the error of the intensity (σI).

[c] $R_{work}=\Sigma|Fo-Fc|/\Sigma|Fo|$, where Fo and Fc are the observed and calculated structure factors, respectively.

[d] R_{free} was calculated using a randomly chosen subset (5%) of the reflections not used in refinement.

Supplementary Table 2. Summary of oligonucleotides used for co-crystallization

DNA	Crystal	Unit cell (Å)	Resolution (Å)	Date
5′-CGATTCAATGAATCCCAAG -3′<br>3′- CTAAGTTACTTAGGGTTCG-5′	Yes	88.2 x 119.6 x 125.7<br>67.7 x 119.6 x 117.9	3.1<br>2.3	10/2018<br>12/2018
5′-CGTATCAATGAATCCCAAG -3′<br>3′- CATAGTTACTTAGGGTTCG-5′	Yes	80.8 x 120.7 x 125.5	3.0	08/2018
5′-CGATTCAATGAATCCCAAG -3′<br>3′- CTAAGTTACTTMGGGTTCG-5′	Yes<br>(M=N6mA)	67.2 x 119.2 x 117.2	2.7	02/2019
5′-CGATCAATGAATCCCAAG -3′<br>3′- CTAGTTACTTAGGGTTCG-5′	No			
5′-CGATTCAATGAATCCCAAG-3′<br>3′-GCTAAGTTACTTAGGGTTC-5′	No			
5′-CGATTCAAGTGAATCCCAA -3′<br>3′- CTAAGTTCACTTAGGGTTG-5′	No			
5′-CGATTCAAGTGAATCCCAA-3′<br>3′-GCTAAGTTCACTTAGGGTT-5′	No			
