# Supplementary material for: The cell cycle-regulated DNA adenine methyltransferase CcrM opens a bubble at its DNA recognition site
Source: Nat Commun. 2019 Oct 10;10:4600. doi: 10.1038/s41467-019-12498-7 (PMC6787082; doi:10.1038/s41467-019-12498-7)
Supplement: Supplementary file 2 — Peer Review File [file 41467_2019_12498_MOESM2_ESM.pdf]

Reviewers' comments:

Reviewer #1 (Remarks to the Author):

In this manuscript, Horton et al present a high-resolution crystal structure of DNA-bound form of the cell cycle regulated DNA methyltransferase (CcrM) enzyme from Caulobacter crescentus. CcrM transfers a methyl group in a SAM (S-adenosyl methionine) dependent manner to N6 position of adenosine base in DNA to maintain the post-replication methylation status. CcrM targets the adenosine base in an asymmetric DNA sequence motif (GANTC) for methylation. Since CcrM exists as homodimer, the question arises how does a dimeric enzyme assemble and act on a single target site. The current CcrM structure reveals unprecedented level of new details and provides a framework to beginning to understand radically different modes of DNA recognition by dimeric DNA MTases. This structure elegantly shows how the two CcrM molecules (A and B) bind to a single target site (GAATC) centrally located within a DNA duplex (18-mer). CcrM contains two structural domains, an N-terminal MTase domain, and a previously uncharacterized c-terminal domain. The CcrM structure reveals that the DNA target specificity comes from the methyltransferase domain of molecule A whereas the c-terminal domain of molecule B contacts non-specifically to the phosphate backbone of the non-targeted DNA strand. Since many eukaryotic MTases work as a dimer, the new findings by Horton et al certainly have broad implications for DNA recognition, specificity and methylation. For example, the extensive distortions in DNA helix spanning 5 base pairs were not observed earlier. This work also attributes new role for the c-terminal domain (CTD) in DNA binding. Authors also note structural similarity between CTD and PWWP domain, found in mammalian cytosine MTase Dnmt3b. Overall, the manuscript is well written and results are presented in extensive depth, details, and with accuracy and clarity. The manuscript deserves publication in Nat Commun.

Major:
None

Minor:

1. Authors note structural similarity b/w CTD and SAND/PWWP. Can they include results of their vector alignment search tool analysis of the CTD, perhaps as supplemental data either as a table or figure showing an overlay of CcrM CTD and PWWP/SAND structures?

2. In the section "Comparison with other dimeric MTases" (on page 11, line 236), authors group CcrM with other M.PvuII and M.RsrI enzymes that act on palindromic sequences. In fact, CcrM recognizes an asymmetric sequence (GANTC), so it should have been grouped with EcoP15I, M.MboIIa).

Reviewer #2 (Remarks to the Author):

Cell cycle-regulated DNA methyltransferase (CcrM)-mediated maintenance N6-adenosine methylation (m6A) within the GANTC sequence provides an important epigenetic mechanism in alpha-proteobacteria. Horton et al reported the crystal structure of CcrM bound to a double-stranded DNA substrate, providing atomic details for the CcrM-substrate interaction. The homodimeric CcrM presents the MTase domain of one monomer to interact with the target strand, while extending the C-terminal domain of the other monomer to interact with the DNA backbone of the non-target strand. Importantly, this protein-DNA interaction leads to a large distortion of DNA conformation, creating a bubble at the target recognition site, which may be implicative of its strong substrate specificity. Overall, this manuscript is well written and provides important insights into CcrM-mediated m6A modifications. I only have a few minor concerns.

1. It will be interesting to know whether a hemimethylated substrate with the same sequence would crystallize under the same condition for structure determination. A previous study (Ref. 6) has suggested that CcrM is more efficient on hemimethylated substrates than unmodified substrates. Based on the current structure, it appears that there is no protein contact with the adenosine ring of A4 on the non-target strand. The authors may want to include discussions on the functional implication of this observation.

2. CcrM shows enzymatic discrimination for ssDNA over ssRNA (Ref. 13). It will be insightful if the authors can model the recognition sequence on the target strand with corresponding ribonucleotides to identify whether any steric clashes would be introduced.

3. The effect of crystallization packing, if any, on the protein/DNA conformations. For example, the observation that the C-terminal domain of CcrM recognizes the DNA backbone of the non-target strand suggests a freedom in its DNA contact. Therefore, it would be important to illustrate the factors (e.g. intermolecular interactions between Mol A and Mol B, domain linker, crystal packing, etc.) that contribute to the current positioning of the C-terminal domain of Mol B.

4. Some of the key protein-DNA interactions, such as A2-recognizing T191 and K193, and G1-recognzing N120, were not labeled in the schematic view of Fig. 1b. On the other hand, some interactions (e.g. V53) labeled in Fig. 1b were not presented in close-up views in Figure 4.

5. The W332A mutation reportedly leads to an inactive enzyme (Ref. 14). This functional importance may not be fully reflected by an interaction with the DNA backbone on the non-target strand. Does it interact with other protein residues (such as R350) of CcrM that may further add to its function?

6. Page 6, line 118-120. The sentence "the interaction…through the DNA strands" needs to be corrected.

7. Page 7, line 140. Change "from where" to "from which".

8. Page 7, line 149. Change "in between" to "in-between".

9. Page 8, line 172-173. ", both of the latter…respectively" need to be reworded.

10. Page 9, line 187. Change "intra-molecule" to "intra-molecular".

Reply-to-editor-and-reviewers:

We are grateful to the editor and two reviewers for their time, effort and very careful examination of our manuscript. We have made changes to the text of the manuscript to address these comments which are in a marked copy of manuscript text.

Specifically, we added three supplementary figures to address (1) the structural similarity between CcrM C-terminal domain and S100β SAND domain, (2) the crystal packing interaction involving the C-terminal domain of molecule B, and (3) a modeling study of ssRNA.

Reviewer #1 (Remarks to the Author):

In this manuscript, Horton et al present a high-resolution crystal structure of DNA-bound form of the cell cycle regulated DNA methyltransferase (CcrM) enzyme from Caulobacter crescentus. CcrM transfers a methyl group in a SAM (S-adenosyl methionine) dependent manner to N6 position of adenosine base in DNA to maintain the post-replication methylation status. CcrM targets the adenosine base in an asymmetric DNA sequence motif (GANTC) for methylation. Since CcrM exists as homodimer, the question arises how does a dimeric enzyme assemble and act on a single target site. The current CcrM structure reveals unprecedented level of new details and provides a framework to beginning to understand radically different modes of DNA recognition by dimeric DNA MTases. This structure elegantly shows how the two CcrM molecules (A and B) bind to a single target site (GAATC) centrally located within a DNA duplex (18-mer). CcrM contains two structural domains, an N-terminal MTase domain, and a previously uncharacterized c-terminal domain. The CcrM structure reveals that the DNA target specificity comes from the methyltransferase domain of molecule A whereas the c-terminal domain of molecule B contacts non-specifically to the phosphate backbone of the non-targeted DNA strand. Since many eukaryotic MTases work as a dimer, the new findings by Horton et al certainly have broad implications for DNA recognition, specificity and methylation. For example, the extensive distortions in DNA helix spanning 5 base pairs were not observed earlier. This work also attributes new role for the c-terminal domain (CTD) in DNA binding. Authors also note structural similarity between CTD and PWWP domain, found in mammalian cytosine MTase Dnmt3b. Overall, the manuscript is well written and results are presented in extensive depth, details, and with accuracy and clarity. The manuscript deserves publication in Nat Commun.

Major: None

Minor:

1. Authors note structural similarity b/w CTD and SAND/PWWP. Can they include results of their vector alignment search tool analysis of the CTD, perhaps as supplemental data either as a table or figure showing an overlay of CcrM CTD and PWWP/SAND structures?

Reply: We added a supplementary Figure S1 to illustrate the structural similarity.

2. In the section “Comparison with other dimeric MTases” (on page 11, line 236), authors

group CcrM with other M.PvuII and M.RsrI enzymes that act on palindromic sequences. In fact, CcrM recognizes an asymmetric sequence (GANTC), so it should have been grouped with EcoP15I, M.MboIIa).

Reply: Agree. We grouped CcrM together with EcoP15I (p11).

Reviewer #2 (Remarks to the Author):

Cell cycle-regulated DNA methyltransferase (CcrM)-mediated maintenance N6-adenosine methylation (m6A) within the GANTC sequence provides an important epigenetic mechanism in alpha-proteobacteria. Horton et al reported the crystal structure of CcrM bound to a double-stranded DNA substrate, providing atomic details for the CcrM-substrate interaction. The homodimeric CcrM presents the MTase domain of one monomer to interact with the target strand, while extending the C-terminal domain of the other monomer to interact with the DNA backbone of the non-target strand. Importantly, this protein-DNA interaction leads to a large distortion of DNA conformation, creating a bubble at the target recognition site, which may be implicative of its strong substrate specificity. Overall, this manuscript is well written and provides important insights into CcrM-mediated m6A modifications. I only have a few minor concerns.

1. It will be interesting to know whether a hemimethylated substrate with the same sequence would crystallize under the same condition for structure determination. A previous study (Ref. 6) has suggested that CcrM is more efficient on hemimethylated substrates than unmodified substrates. Based on the current structure, it appears that there is no protein contact with the adenosine ring of A4 on the non-target strand. The authors may want to include discussions on the functional implication of this observation.

Reply: We did perform the crystallization with hemi-methylated DNA and got crystals under similar conditions. However, the structure did not show any difference with unmodified DNA. We added a statement in the Methods section (p15). This observation illustrates that CcrM has about equal activity (<2-fold) on both unmodified and hemimethylated substrates in vitro.

2. CcrM shows enzymatic discrimination for ssDNA over ssRNA (Ref. 13). It will be insightful if the authors can model the recognition sequence on the target strand with corresponding ribonucleotides to identify whether any steric clashes would be introduced.

Reply: Yes, we modeled ssRNA. The additional 2'-hydroxyl group (OH) onto sugar ribose (as happens in RNA) potentially results in intra-strand repulsion. We added a supplementary Figure S3.

3. The effect of crystallization packing, if any, on the protein/DNA conformations. For example, the observation that the C-terminal domain of CcrM recognizes the DNA backbone of the non-target strand suggests a freedom in its DNA contact. Therefore, it would be important to illustrate the factors (e.g. intermolecular interactions between Mol A and Mol B, domain linker, crystal packing, etc.) that contribute to the current positioning of the C-terminal domain of Mol

B.

Reply: The C-terminal domain of molecule B is indeed involved in the crystal packing contacts with two neighboring molecules. We provided supplemental figure S2 to illustrate the interaction.

4. Some of the key protein-DNA interactions, such as A2-recognizing T191 and K193, and G1-recognzing N120, were not labeled in the schematic view of Fig. 1b. On the other hand, some interactions (e.g. V53) labeled in Fig. 1b were not presented in close-up views in Figure 4.

Reply: We added the base-interacting residues T191, K193, and N120 in Figure 1b. The water-mediated phosphate-interaction with the main chain atom of residue V53 is illustrated in the schematic of Fig. 1b.

5. The W332A mutation reportedly leads to an inactive enzyme (Ref. 14). This functional importance may not be fully reflected by an interaction with the DNA backbone on the non-target strand. Does it interact with other protein residues (such as R350) of CcrM that may further add to its function?

Reply: The reviewer is correct. W332 is sandwiched between R350 and I316 via van der Waals contacts, providing additional support for the local stability. We added a sentence on p10.

6. Page 6, line 118-120. The sentence “the interaction…through the DNA strands” needs to be corrected.
Reply: We deleted three words “the interaction between”.

7. Page 7, line 140. Change “from where” to “from which”.
Reply: Done.

8. Page 7, line 149. Change “in between” to “in-between”.
Reply: Done.

9. Page 8, line 172-173. “, both of the latter…respectively” need to be reworded.
Reply: We split the long sentence into two shorter sentences.

10. Page 9, line 187. Change “intra-molecule” to “intra-molecular”.
Reply: Done.
